# Targeted activation of Stat3 in combination with paclitaxel results in increased apoptosis in epithelial ovarian cancer cells and a reduced tumour burden

**DOI:** 10.1111/cpr.12719

**Published:** 2019-11-28

**Authors:** Hongyi Li, Yanping Qian, Xi Wang, Ruyu Pi, Xia Zhao, Xiawei Wei

**Affiliations:** ^1^ Department of Gynecology and Obstetrics Development and Related Disease of Women and Children Key Laboratory of Sichuan Province Key Laboratory of Birth Defects and Related Diseases of Women and Children Ministry of Education West China Second Hospital Sichuan University Chengdu China; ^2^ Lab of Aging Research and Nanotoxicology State Key Laboratory of Biotherapy West China Hospital Sichuan University and Collaborative Innovation Center Chengdu China

**Keywords:** apoptosis, epithelial ovarian cancer, Napabucasin (BBI608), paclitaxel, Stat3 inhibitor

## Abstract

**Objectives:**

Stat3 is persistently activated in ovarian cancer cells, with a crucial role in tumour onset and progression. In this study, we examined the anti‐tumour effect of a small‐molecule inhibitor napabucasin (BBI608) on epithelial ovarian cancer (EOC) in vitro and in vivo, and investigated the underlying molecular mechanism of this drug in combination with paclitaxel.

**Materials and Methods:**

A total of 156 ovarian cancer patient samples were analysed to determine the correlation between pStat3 expression in tumour cells and the prognosis of EOC patients. The anti‐tumour effect of BBI608 and/or paclitaxel on ovarian cancer in vitro was evaluated by CCK‐8, flow cytometry, Western blot and transwell assays. An in vivo intraperitoneal model was performed to confirm the effect of BBI608 on pStat3‐mediated peritoneal metastasis when combined with paclitaxel.

**Results:**

Patients with high expression of pStat3 had poorer overall survival and progression‐free survival than those with low pStat3 expression. The synergy of BBI608 in combination with paclitaxel exerted dramatic growth inhibition and induced apoptosis in EOC cell lines. In vivo, the combination of two drugs significantly decreased intraperitoneal tumour burden and ascites volume, prolonged survival of tumour‐bearing mice compared with each monotherapy; these results were associated with downregulation of phospho‐Stat3 and activation of apoptosis pathway.

**Conclusions:**

Targeting the activation of Stat3 may be a potential therapeutic approach for EOC by acting synergistically with paclitaxel.

## 1 INTRODUCTION

Epithelial ovarian cancer (EOC) refers to a gynaecologic malignancy exhibiting the largest lethality in the female population,^1^ and doctors could not diagnose most cases with EOC until the disease displays late stage, noticeably promoting relapse and early death.^2^ The ineffective EOC prognosis is primarily attributed to subtle symptoms in ovarian cancer's early stages, resulting in delayed diagnosis and easy transmission of the disease through peritoneal transplantation and blood dissemination.^3^

The current standard for the management of advanced EOC consists of surgical debulking and adjuvant chemotherapy with a dose‐dense taxane (paclitaxel) and platinum (cisplatin or carboplatin) regimen, resulting in initial remission in up to 80% of patients.^4^ This treatment regimen results in a significant reduction of tumour burden by inducing cell death through an apoptotic pathway, as well as causing cell cycle arrest at G2/M phase. Unfortunately, the majority of these patients eventually experience tumour relapse within two years because EOC cells either are less sensitive or become resistant to anti‐cancer drugs after consecutive therapy, resulting in a 5‐year survival rate as low as ~30%.^5^, ^6^ For these reasons, treatment for EOC remains challenging, and there is an urgent need to develop more feasible agents exhibiting low toxicity with more distinct molecular target in order to effectively assist in the treatment of ovarian cancers.

Signal transducer and activator of transcription 3 (Stat3) belongs to the Stat family that mediates cellular responses to specific cytokines and growth factors [interleukin‐6 (IL‐6), epidermal growth factor (EGF), granulocyte colony‐stimulating factor (G‐CSF), leukaemia inhibitory factor (LIF), etc] by regulating downstream genes expression. It has been considered the most frequently correlated with tumorigenesis and is a promising molecular target for cancer therapies.^7^ While its activation is transient under physiological conditions, Stat3 becomes continuously activated in a high proportion of solid and haematopoietic malignancies (including breast, ovarian and prostate cancer), thus contributing to malignant transformation and progression. For example, activated Stat3 can bond to DNA, access into the nucleus and stimulate the transcription of various genes that regulate important function of cells, including maintenance of cell survival, uncontrolled cellular proliferation, promotion of angiogenesis and facilitation of resistance to apoptosis induced by conventional chemotherapy.^7^ A recent study showed that infiltrating macrophages significantly enhanced Stat3 activation in drug‐resistant recurrent ovarian tumours compared to matched primary tumours. In addition, recent researches suggested that an elevated level of pStat3 displays relations to poor prognosis of many cancers, including lung cancer,^8^ colorectal cancer^9^ and lung cancer.^10^ Stat3’s critical role in tumorigenesis and tumour prognosis emphasizes that novel anti‐cancer drugs capable of regulating Stat3‐mediated signalling events in a negative manner are urgently required. Although several reasonable ways of targeting pStat3 function and several targeted inhibitors have been reported, the role of Stat3 in the pathogenesis of ovarian cancer has not been fully elucidated, and so far, no small molecular inhibitor of Stat3 has been prepared for clinical development.

Napabucasin (also called BBI608) is a newly discovered, orally administered small molecule that can specifically inhibit gene transcription of Stat3. This compound is currently in clinical development as a treatment for a diverse range of cancers (eg pancreatic cancer,^11^ lung cancer^12^ and pancreatic cancer).^13^ As an attempt to develop alternative treatments, we investigated the anti‐tumour effect and molecular mechanism of BBI608 on ovarian carcinoma in vivo and in vitro. We also demonstrated the potency of this compound when combined with the traditional chemotherapeutic drug paclitaxel. In this study, Stat3’s overactivation and overexpression in tumour tissues of EOC patients were observed, and we speculated that the persistent stimulation made by the Stat3 signalling pathway is likely to be vital to EOC prognosis. Our experimental results demonstrated that the Stat3 inhibitor BBI608 not only inhibited cell proliferation, colony‐forming ability, cell invasion and cell migration as well as increased cell apoptosis in vitro but also significantly enhanced the anti‐tumour effect of paclitaxel. Subsequent, in vivo experimental results showed that the combination of BBI608 and paclitaxel could decrease tumour burden and ascites volume better than monotherapy by blocking the activation of Stat3 and increasing cleaved caspase‐3‐positive cells’ number without detectable toxicity in a peritoneal metastasis mouse model. Taken together, these data emphasize that BBI608 may offer therapeutic benefits against advanced epithelial ovarian cancer and the need to further explore the role of BBI608 combined with chemotherapy in pre‐clinical ovarian cancer models.

## 2 MATERIALS AND METHODS

### 2.1 Patient samples

Shanghai Outdo Biotech (National Engineering Centre for Biochip at Shanghai) provided samples from primary EOC of 160 females, and the ethics committee (permit number: YBM0502) gave approval of the application of chart review and tissue blocks. The average follow‐up time reached 84 months (60‐108 months), the mean age of 156 assessable cases reached 50.6 years (23‐73 years), and the total survival rate in 5 years reached 55.2%. World Health Organization criteria guided histopathologic diagnosis; 74.4% of cases were serous papillary cystadenocarcinoma, 14.1% were mucinous cystadenocarcinoma, 7.7% were endometrioid carcinoma, 2.6% were clear cell carcinoma, and 1.2% were mixed cystadenocarcinoma. We obtained the disease‐specific survival rate as the per cent of cases having come through the disease for a specific period; it was found as the time after treatment or disease diagnosis, and we counted the number of deaths from EOC only. The specimens were stained with anti‐pStat3 antibody (CST, #9145S), and the percentage of cells positive for pStat3 nuclear staining was calculated by reviewing the entire areas of each tissue and was described as follows: low, 0‐50% positive cells; and high, ≥50% positive tumour cells.^14^, ^15^ For the analysis of staining results, two observers blinded to the patients' information and independently scored and grouped these specimens according to the nuclear expression of pStat3 in tumour cells.

### 2.2 Cell culture and animals

The American Type Culture Collection (ATCC, Rockville, MD, USA) provided human ovarian cancer cell lines A2780 and SKOV3 and mouse ovarian cancer cell line ID8. These cells were propagated in a humidified incubator in Dulbecco's modified Eagle's medium (DMEM) containing 10% foetal bovine serum (FBS; Gibco) and 1% antibiotics (penicillin and streptomycin) at 37°C with 5% CO2. The experimental animals used in this study were C57 BL/6 mice (6‐ to 8‐week‐old) and female BALB/c mice (4‐ to 6‐week‐old) provided by Beijing HFK bioscience Co. Ltd. (Beijing, China). Protocols for the use of animals in these studies were reviewed and approved by the Institutional Animal Care and Use Committee of Western China Second Hospital.

### 2.3 Reagents and antibodies

BBI608 was provided by the Selleck Express (Houston, TX, USA). By high‐performance liquid chromatography (HPLC) analysis, purity (99.18%) was ascertained. In each vitro study, BBI608 was initially produced as a 10 mM stock solution in dimethyl sulfoxide (DMSO) and incubated at −80°C. Subsequently, 0.1% DMSO acted as a vehicle control, and the stock solution was deliquated with relevant assay medium. For in vivo experiments, BBI608 was prepared in 1% (w/v) carboxymethyl cellulose sodium solution (1% NaCMC) and dosed at 0.1 ml/10 g of body weight. Paclitaxel was purchased from Meilunbio with purity >99%. Sigma Chemical Co. provided Cell Counting Kit‐8 (CCK‐8), DMSO and NaCMC. BD Biosciences provided the Annexin V‐FITC Apoptosis Detection Kit. Cell Signaling Technology provided the primary antibodies against Stat3/pStat3‐Tyr705, Bcl‐2, Bax, Mcl‐1, cleaved caspase 3, GAPDH and mouse monoclonal anti‐Ki67.

### 2.4 Cell viability assay

The viability of BBI608 alone or in combination with paclitaxel in ovarian cancer cells was analysed by the CCK‐8 assay following the instructions of the producer. Briefly, A2780, ID8 and SKOV3 cells underwent seeding process in a final volume of 100 μL in a 96‐well plate at 2 ~ 4 × 10^3^ cells/well density and grown for 24 hours. After adding different concentrations of BBI608 compounds (0, 0.1, 0.25, 0.5, 1, 2, 4, 6, and 8 µmol/L), the cells were further cultured for another 24 hours. Then, we dropped 10 µL of diluted CCK‐8 regent in every well, and the culture plate underwent incubation as shielded from light for 2 ~ 4 hours at 37°C. With the use of a microplate instrument, we ascertained optical density (OD) value at a wavelength of 450 nm. Using the median‐effect model of Chou‐Talalay and with CompuSyn software, we ascertained the combination index (CI).^16^ CI < 1 indicates synergism. Each experiment was carried out in triplicate; we obtained the half inhibitory concentration (IC50) with Prism 7 (GraphPad software).

### 2.5 Morphological analysis by Giemsa staining

For directly identifying the anti‐proliferation influence of BBI608 and its anti‐tumour effect when used with 1nM paclitaxel, we analysed the SKOV3 cells by Giemsa stain assay. Briefly, SKOV3 cells (2 × 10^5^ cells/well) underwent plating process in a 6‐well plate for 12 hours. After following 24 hours of drug treatment, the cells underwent cleaning process with cold phosphate‐buffered saline (PBS) and fixing process for 15 minutes with 4% paraformaldehyde solution. Subsequently, the cells underwent cleaning process with PBS twice, incubation in Giemsa solution (Solarbio) for 10‐15 minutes following the directives of the producer and then PBS washing. Afterwards, the results of cell staining were observed by an inverted microscope (Leica, DM4000B) with 400 × amplification.

### 2.6 Colony formation assay

SKOV3 cells underwent seeding process at 1000 cells/well on six‐well plates. After cultured at 37°C for 12 hours, the cells underwent the treatment with BBI608 at different concentrations and the culturing process for 14 days. The cells were then briefly cleaned with PBS and kept stationary with cold methanol for 10 minutes and then cleaned with PBS 3 times. Crystal violet was used to stain the cells for 10 minutes at ambient temperature. After that, the cells underwent washing process 3 times with PBS and air‐dried at room temperature. The number of colonies was counted using a microscope. Data presented denote the average of 3 separated experiments.

### 2.7 Cell apoptosis assay by flow cytometry

For verifying the induction of apoptosis in ovarian cancer cells, Annexin V‐FITC apoptosis detection kit was performed after BBI608 or/and paclitaxel (1 nM) treatment. Briefly, A2780 and SKOV3 cells were disassociated and plated in 6‐well plates at 2 × 10^5^ cells/well and treated with BBI608 at increasing concentrations (A2780: 0, 0.2, 0.3, 0.4, 0.5 µmol/L; SKOV3: 0, 0.25, 0.5, 1, 2 µmol/L). After incubated at 37°C for 24 hours, we harvested the cells and cleaned them 2 times with cold PBS. With the use of the Annexin V‐FITC apoptosis detection kit (first stained with Annexin V for 10 minutes, subsequently PI introduced to stain cells for 5 minutes), we ascertained apoptosis levels by flow cytometry. We analysed the data using NovoExpress software.

### 2.8 Western blot analysis for EOC cell lines

Briefly, cells were treated with BBI608 or/and paclitaxel for 24 hours at the designed concentration and subsequently cleaned two times with cold PBS and lysed through the addition of 1 × SDS sample buffer (100 µL per well of 6‐well plate). Next, to complete cell lysis and shear DNA, the cells underwent sonication 10‐15 seconds in sample loading buffer and denatured in a boiling water bath (95‐100°C for 10 minutes; cool on ice). With the use of the Pierce™ Rapid Gold BCA Protein Assay Kit (Thermo Fisher, Catalogue # A53225), we ascertained protein concentrations to ensure equal loading. Total protein from each sample was run through 12.5% sodium dodecyl sulphate‐polyacrylamide gel electrophoresis (SDS‐PAGE) gels and transferred onto polyvinylidene difluoride (PVDF) membranes. After electrophoresis, the membranes underwent the blocking process for 2 hours by 5% bovine serum albumin (BSA) at 37°C and subsequently the incubation overnight at 4°C with specific primary antibodies. When three times of washing with Tris‐buffered saline and 0.1% Tween‐20 (TBST) was finished, the membranes underwent incubation for 1 hours with secondary antibodies at room temperature. With a commercially available optimized chemiluminescence detection kit (Bio‐Rad Laboratories), we imaged the bands. We analysed all immunoreactive bands’ density with ImageJ software.

### 2.9 Wound healing assay

For ascertaining the migrative ability of ovarian tumour cells, a wound healing assay was performed. SKOV3 cells underwent the seeding process in a 24‐well culture dish and grown to 80%‐90% fusion. With a 2‐mm‐wide sterilized plastic pipette tip, a scratch was made, and the debris was washed with aseptic PBS. After treatment with different concentrations of BBI608, we removed the excess liquid in the wells, and the cells underwent the cleaning process using sterilized PBS and covering process using serum‐free medium. Migration of cells into the wound area was then observed at different times (0 and 24 hours). Cells migrated into or protruded from the edge of the wound were observed and photographed with the use of an inverted microscope at various times. Four regions were randomly selected in each well at 100 × magnification, and in each experiment, 3 wells of each group underwent quantifying process.

### 2.10 Boyden chamber migration and invasion assay

For the assessment of the migration and invasion abilities of tumour cells in transwell insert chambers (8.0 µm; Corning Incorporated), we performed migration and Matrigel invasion assays. We introduced 600 μL of medium supplemented by 30% FBS to the lower chamber. Subsequently, in the upper chamber at 50 000 cells/well in 200 µL of serum‐free medium, cells in the presence of BBI608 (A2780: 0, 0.1, 0.2, and 0.3 µM; SKOV3: 0, 0.25, 0.5, 1.0 µM) underwent seeding process. After 24 hours of cell migration, the cells on the lower surface of chamber underwent the fixing process in 4% paraformaldehyde and then the staining process with 0.5% crystal violet; in the meantime, we removed the cells on the upper chamber with a cotton swab. We counted and photographed migratory cells using a light microscope (Nikon Corporation) at 200 × amplification. We conducted the invasion assay given a published study.^17^ In brief, the transwell chamber's upper surface underwent the pre‐coating process with 60 µL of diluted Matrigel (BD Biosciences). When the Matrigel polymerization was achieved, the bottom chamber was full of 600 µL of culture medium supplemented by 30% FBS. We added 200 µL of serum‐free medium containing A2780 or SKOV3 cells to each transwell upper chamber and treated it with BBI608 at various concentrations. When a 24‐hour incubation was achieved, we removed non‐invading cells on the upper surface and fixed the invading cells in the lower surface of the chamber with cold 4% paraformaldehyde and stained them with 0.5% crystal violet. Subsequently, with the use of an optical microscope, we calculated and imaged the invading cells. The percentage of migratory or invading cells suppressed by BBI608 underwent quantification.

### 2.11 Quantitative RT‐PCR (qRT‐PCR)

With the use of an RNA Extraction Kit (TIANGEN BIOTECH, DP419), overall RNA was harvested, following the directives of the producer; with the use of the SuperScript III Reverse Transcriptase Kit (Invitrogen), it subsequently underwent reverse transcription into cDNA. qPCR was performed using SYBR Select Master Mix (Invitrogen) in StepOnePlus PCR System with specific gene primers. Templated cDNA was first denatured at 95°C for 1 minute, and the primers were specifically annealed for 1 minute and then extended at 72°C for 1 minute. Cycles then underwent elongation step of 72°C for 10 minute. Each sample was repeated three times, and the target gene expression level was normalized to GAPDH and analysed using the 2^−ΔΔCt^ method. Primer sequences used were seen in Table S1.

### 2.12 In vivo anti‐tumour study

The Institutional Animal Care and Treatment Committee of Sichuan University in China (Permit Number: 2 018 028) approved and performed all animal experiments. For xenograft studies, SKOV3 cells were subcutaneously injected into immunosuppressed BALB/c mice (1 × 10^7^ cells per mouse) and allowed to form subcutaneous tumours. ID8 cells were inoculated intraperitoneally into female C57BL/6 mice (5 × 10^6^ cells per mouse) and allowed to form abdominal tumours. Approximately five days after tumour cell inoculation, the tumour‐bearing immunosuppressed BALB/c mice underwent the randomization process in 3 groups (6 mice per group) and then intragastric (i.g.) administration of BBI608 40 mg/kg/d, vehicle or control for 21 days, respectively. We obtained tumour size according to the formula: tumour volume (mm^3^) = 0.52 × L × W^2^, where L denotes the length and W indicates the width. We recorded body weight and tumour volumes per 3 days. For the abdominal metastatic model, injection of cancer cells is designated as day 0, and treatment was initiated at day 10. C57BL/6 tumour‐bearing mice were administered 12 mg/kg paclitaxel (intraperitoneally) once, 40 mg/kg BBI608 (orally) or vehicle daily (6 consecutive days, followed by a one‐day dose holiday) or the combination of the two drugs. Animals were sacrificed when tumour size reached approximately 1000 mm^3^ or the mice weight increased by 50%. After all animals received euthanasia by cervical dislocation, the lung, liver, heart, kidney and spleen of each mouse were harvested for haematoxylin and eosin (H&E) staining. In addition, the biochemical analysis on blood for every mouse was also performed to evaluate drug toxicity.

### 2.13 Immunohistochemistry staining and haematoxylin and eosin staining

The tumour tissues first underwent the fixing process in 4% paraformaldehyde at ambient temperature and the embedding process in paraffin. Paraffin‐embedded tissues were cut (4 µm thick), and subsequently, the samples were dewaxed, hydrated and incubated with the set antibodies (pStat3, Ki67 and cleaved caspase‐3) throughout the night at 4°C. By relevant secondary antibodies, the staining signal was ascertained. Subsequently, these slides underwent the staining process with diaminobenzidine and the counterstaining process using haematoxylin. Using Image‐Pro Plus 6.0 software, quantitative determination of immunochemical data was performed. The heart, liver, spleen, lung and kidney tissues of the vehicle and BBI608 group were kept stationary in 4% formaldehyde and embedded in paraffin to achieve histological analysis. Paraffin organ tissue sections (5 µm thick) received the deparaffinization and rehydration, and subsequently, they underwent the staining process with haematoxylin and eosin. With the use of an optical microscope (400× amplification, Leica, DM4000B), we took images.

### 2.14 TUNEL staining and Western blot analysis for EOC tumour tissues

For TUNEL assays, paraffin‐embedded mouse tumour tissues underwent the cutting process and then the staining process following the directives of the producer (Promega G7130). By Leica DM2500 fluorescence microscope, we took the images.

For Western blot analysis, EOC tumour tissues (60‐80 mg) preserved with liquid nitrogen from vehicle and experimental mice were cut up and incubated for 30 minutes in 0.5 mL of T‐PER Tissue Protein Extraction Reagent on ice. Subsequently, the lysed tissue underwent centrifugation for 15 minutes at 4°C at 15,000 × g. The proteins were isolated by 12.5% SDS‐PAGE electrophoresis, electro‐transferred onto nitrocellulose membranes, blocked with 5% skim milk, and probed overnight with anti‐PARP, pStat3, Stat3, caspase‐3 and cytochrome C antibodies at 4°C. The membranes underwent the cleaning process and then the exposure to HRP‐conjugated secondary antibody for 1 to 2 hours, and lastly the detection with a chemiluminescent substrate.

### 2.15 Statistical analysis

Data represented as mean ± standard error of three separated experiments. By GraphPad Prism 7.0, we performed all statistical analyses. Student's *t* test and one‐way analysis of variance were conducted for analysing the differences between data sets. Statistically noticeable *P* values were labelled as: *P < .05, **P < .01, ***P < .001, ****P < .0001. A probability level of below 0.05 (*P* < .05) was regarded as a significant difference. We employed the Kaplan‐Meier approach to achieve survival analysis. For calculating the significance of diversifications in the survival analysis, a log‐rank test was performed.

## 3 RESULTS

### 3.1 **Phosphorylated‐Stat3 (pStat3**) **is upregulated in tumour cells and indicates poor prognosis in ovarian cancer**

**Figure 1:**
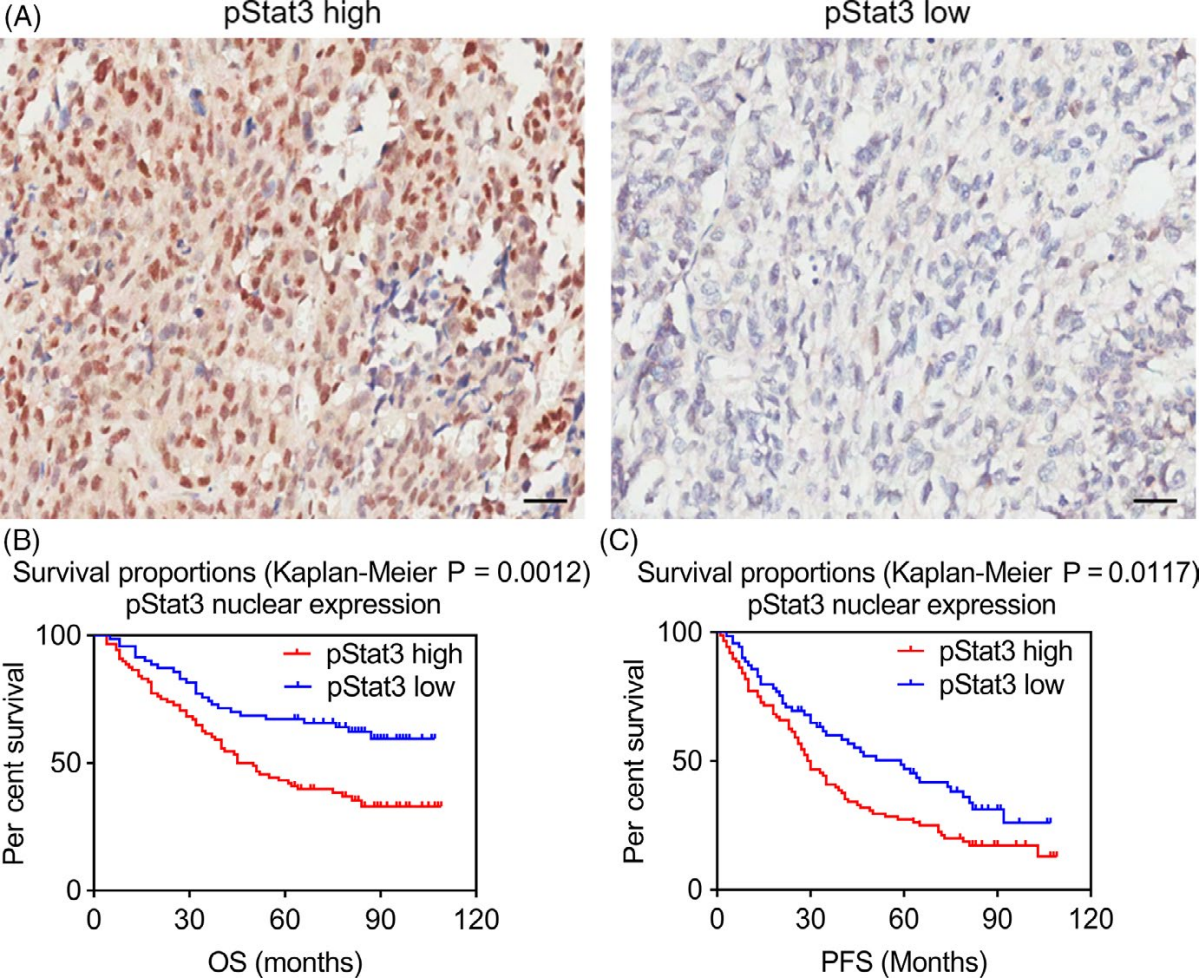
Survival curves of ovarian cancer patients grouped by nuclear pStat3 expression in EOC tissues. (A) Immunohistochemistry images with labelled pStat3 high/low were representative regions of pStat3 expression in ovarian tumour microarray (magnification, ×200). (B) and (C) Association of pStat3 expression with the patients’ overall survival (OS) and progression‐free survival (PFS) in EOC, respectively

Through the review of medical records, we upgraded follow‐up information in July 2018. We made a successful follow‐up of a total of 156 EOC patients, among whom 85 died. The results from pStat3 staining based on clinicopathological characteristics are presented in Table 1. Overall survival and progression‐free survival were compared according to nuclear expression of pStat3^high^ (with >50% of nuclei stained) and pStat3^low^ (<50% of nuclei stained) protein (Figure 1A). The results indicated that females who had tumours with high pStat3 expression (n = 86) had poorer survival than females carrying tumours with low pStat3 expression (n = 70) (Figure 1B; log‐rank test, χ^2^ = 10.57, *P* < .01). pStat3^high^ patients’ mean survival time reached 51.23 ± 3.36 months, which was noticeably less than pStat3^low^ patients’ (74.52 ± 3.72 months). The patients with high expression of pStat3 were associated with poor PFS in comparison with patients with low pStat3 expression (Figure 1C; log‐rank test, χ^2^ = 6.354, *P < .05).

**Table 1:** Relationship between tyrosine‐activated Stat3 (pStat3) immunoreactivity of tumour and pathological features of human epithelial ovarian carcinoma

Characteristic	n (%）	pStat3 nuclear expression		χ^2^	*P* value
		Low	High		
Age (y)	156			2.988	0.084
≤50	64 (41.03)	34	30		
>50	92 (58.97)	36	56		
Tumour grade	156			0.943	0.624
G1	27 (17.3)	11	16		
G2	37 (23.7)	18	19		
G3	84 (53.8)	33	51		
Disease stage	156			3.299	0.348
Stage I	9 (5.8)	5	4		
Stage II	37 (23.7)	17	20		
Stage III	78 (50.0)	38	40		
Stage IV	32 (20.5)	10	22		
Histotype	156			8.045	**0.045**
Serious carcinoma	116 (74.4)	46	70		
Mucinous adenocarcinoma	22 (14.1)	14	8		
Endometrioid adenocarcinoma	12 (7.7)	5	7		
Other subtypes	6 (3.8)	5	1		
Diameter of primary focus (cm)	156			6.332	**0.012**
<10 cm	61 (39.1)	35	26		
≥10 cm	95 (60.9)	35	60		
Lymph node metastasis	156			2.394	0.122
N0	113 (72.4)	55	58		
N1	43 (27.6)	15	28		
Distant metastasis	156			0.180	0.672
M0	111 (71.2)	51	60		
M1	45 (28.8)	19	26		
Progressive disease	156			9.441	**0.002**
No recurrence	72 (46.2)	37	35		
Recurrence	84 (53.8)	23	61		

Note

Boldface indicates *P* < .05.

### 3.2 BBI608 effectively inhibits EOC cell proliferation and colony formation ability and increases drug sensitivity of EOC cells to paclitaxel

**Figure 2:**
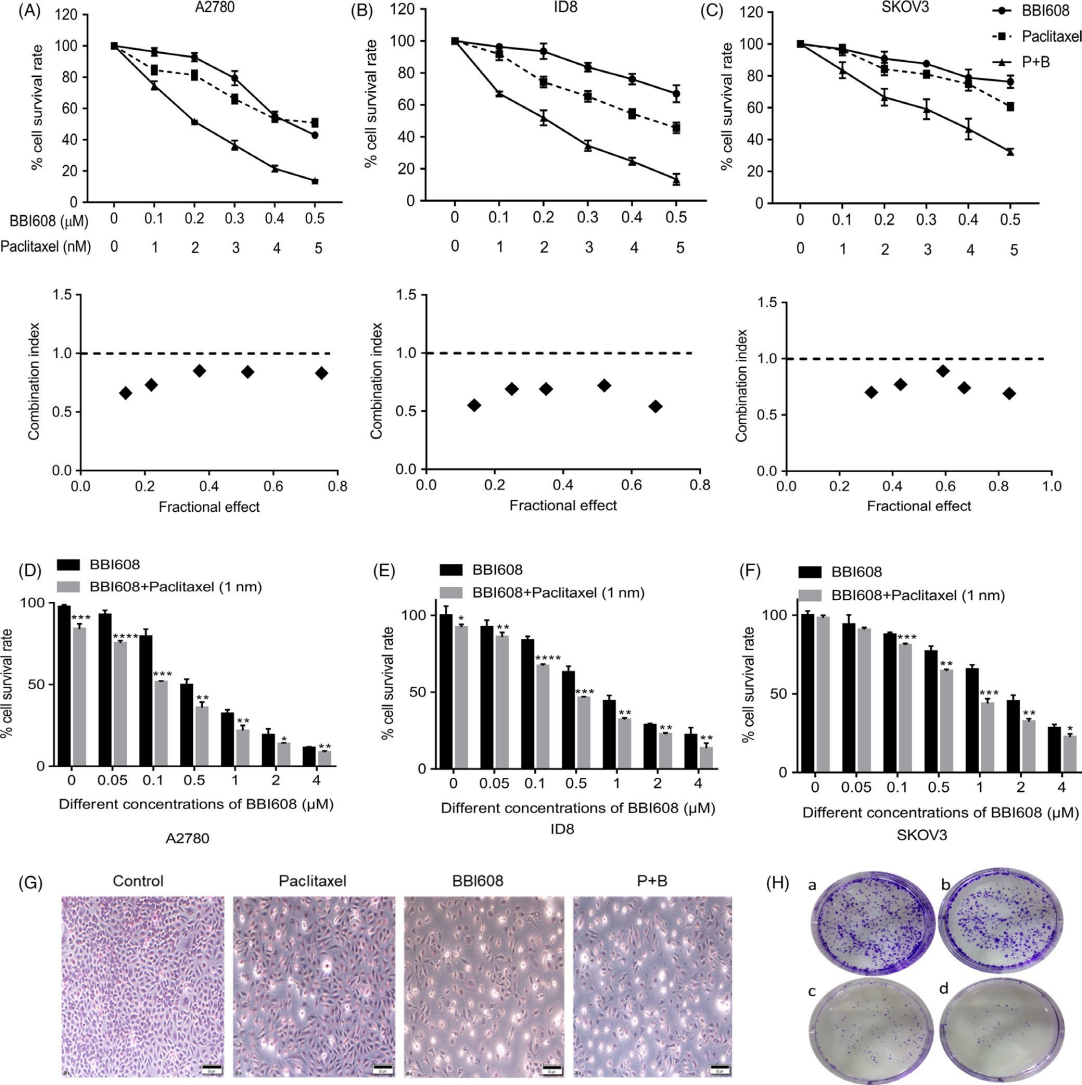
BBI608 acted synergistically with paclitaxel in inhibiting EOC cell proliferation and colony formation ability. (A), (B) and (C) EOC cells A2780, ID8 and SKOV3 were treated with various concentrations of BBI608 or paclitaxel alone or their combination for 24 h, and then, the cell viability was analysed by CCK‐8 assay. The combination index (CI) was determined using the Chou‐Talalay Method. CI < 1 indicates that the interaction between BBI608 and paclitaxel was synergistic. (D), (E) and (F) The proliferation of A2780, ID8 and SKOV3 cells treated with different concentrations of BBI608 and combined paclitaxel and BBI608 for 24h. The data shown are the means ± SD of a representative experiment performed in triplicate (n = 3). (G) Representative images of Giemsa stain in SKOV3 cell line after drug treatment (magnification, ×400). (H) Representative images of colony formation assay in SKOV3 cell line after drug treatment. *P < .05; **P < .01; ***P < .001*; ***P < .001

Previous studies demonstrated that in vitro treatment of EOC cell lines with cisplatin or paclitaxel led to the activation of the JAK2/STAT3 pathway.^18^, ^19^ EOC cells appear resistant to chemotherapy due to elevated activation of Stat3.^20^ Therefore, we examined whether targeting pSta3 levels with BBI608 could sensitize EOC cells to paclitaxel. Indeed, we found that subcytotoxic combinations of BBI608 and paclitaxel‐induced synergistic cell death in all three EOC cells (A2780, ID8 and SKOV3) tested (Figure 2A‐C, CI < 1).

Then, we extended our investigations to effect of a low concentration paclitaxel combining with different concentrations of BBI608 on EOC cells. The anti‐proliferative activity of BBI608 against the EOC cell lines A2780, ID8 and SKOV3 was assessed by the CCK‐8 cytotoxicity assay. When exposed to BBI608 for 24 h, the IC50 of BBI608 in A2780, ID‐8 and SKOV3 cells was 0.4834, 0.7113 and 1.4470 µM, separately. As shown in Figure 2D, 2 and 2, BBI608 inhibited cell proliferation in a manner relying on concentration. Furthermore, cell proliferation inhibition was significantly increased when BBI608 was combined with a low dose of paclitaxel (1nM). The IC50 values of BBI608 in A2780, ID‐8 and SKOV3 cells after being treated for 24 hours were 0.2796, 0.4603 and 0.7343 µM, respectively (Figure S1). SKOV3 cell morphology was also observed by Giemsa staining. As shown in Figure 2G, in contrast to the control group, the different treatment groups showed a decreased number of cells, wrinkled morphology and increased cellular surface brightness. To further verify whether BBI608 could inhibit SKOV3 cell proliferation, we performed colony formation assays after BBI608 (1 µM) and/or paclitaxel (1 nM) treatment. Clonogenic assays noticeably suggested that the clone formation of SKOV3 cells was reduced in the combination group after exposure to drug for 24 hours (Figure 2H: a, control; b, BBI608; c, paclitaxel; d, P + B). Furthermore, the number of colonies in the co‐treatment group (BBI608 and paclitaxel) was remarkably less than the other groups (Figure S2). Therefore, these results indicated that BBI608 could suppress ovarian cancer cells’ proliferation in a concentration‐dependent manner and could increase the sensitivity of EOC cells to paclitaxel.

### 3.3 BBI608 specifically inhibits constitutive Stat3 activation in A2780 and SKOV3 cells and induces apoptosis in a concentration‐dependent manner

**Figure 3:**
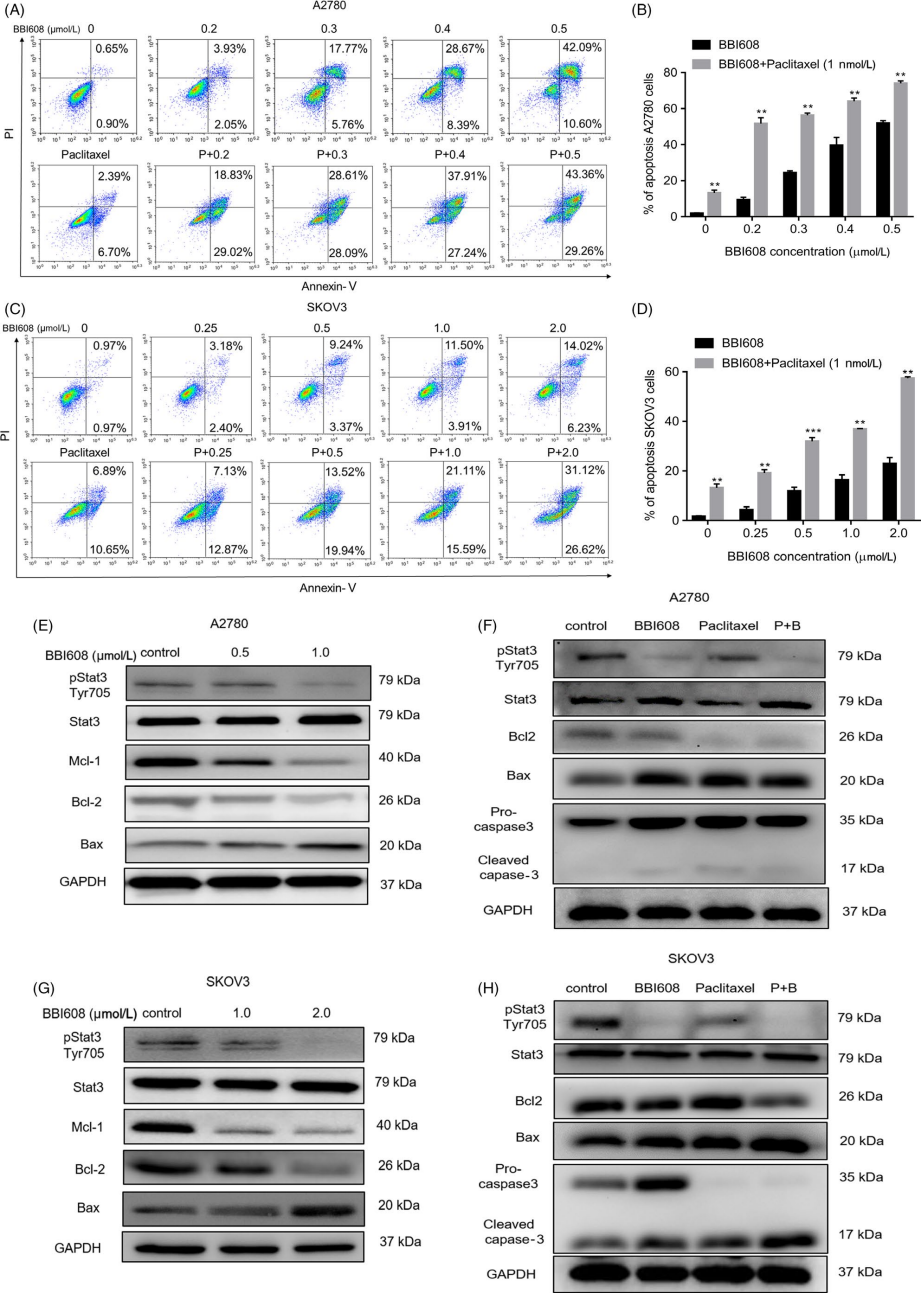
BBI608 induces A2780 and SKOV3 ovarian cancer cells apoptosis. (A) A2780 and (C) SKOV3 ovarian cancer cells were treated with BBI608/BBI608 + paclitaxel (1nM) at indicated doses for 24h, and the level of apoptosis was evaluated using the Annexin V/PI dual‐labelling technique, as determined by FCM. Statistic results of apoptosis assays, (B) A2780 and (D) SKOV3 cells positive for both Annexin V and PI were considered apoptotic. Data are expressed as means ± SD from three independent experiments. (E) and (F) Western blot analyses of ovarian cancer cells (A2780 and SKOV3) treated with different concentrations of BBI608 for 12 h to evaluate protein expression of pStat3, Stat3, Bcl‐2, Bax and Mcl‐1. GAPDH was employed as a standard. (G) and (H) Equal amounts of lysates were analysed by Western blot analysis using antibodies against pStat3, Stat3, Bcl‐2, Bax and caspase‐3. GAPDH was employed as a standard. *P < .05; **P < .01

**Figure 4:**
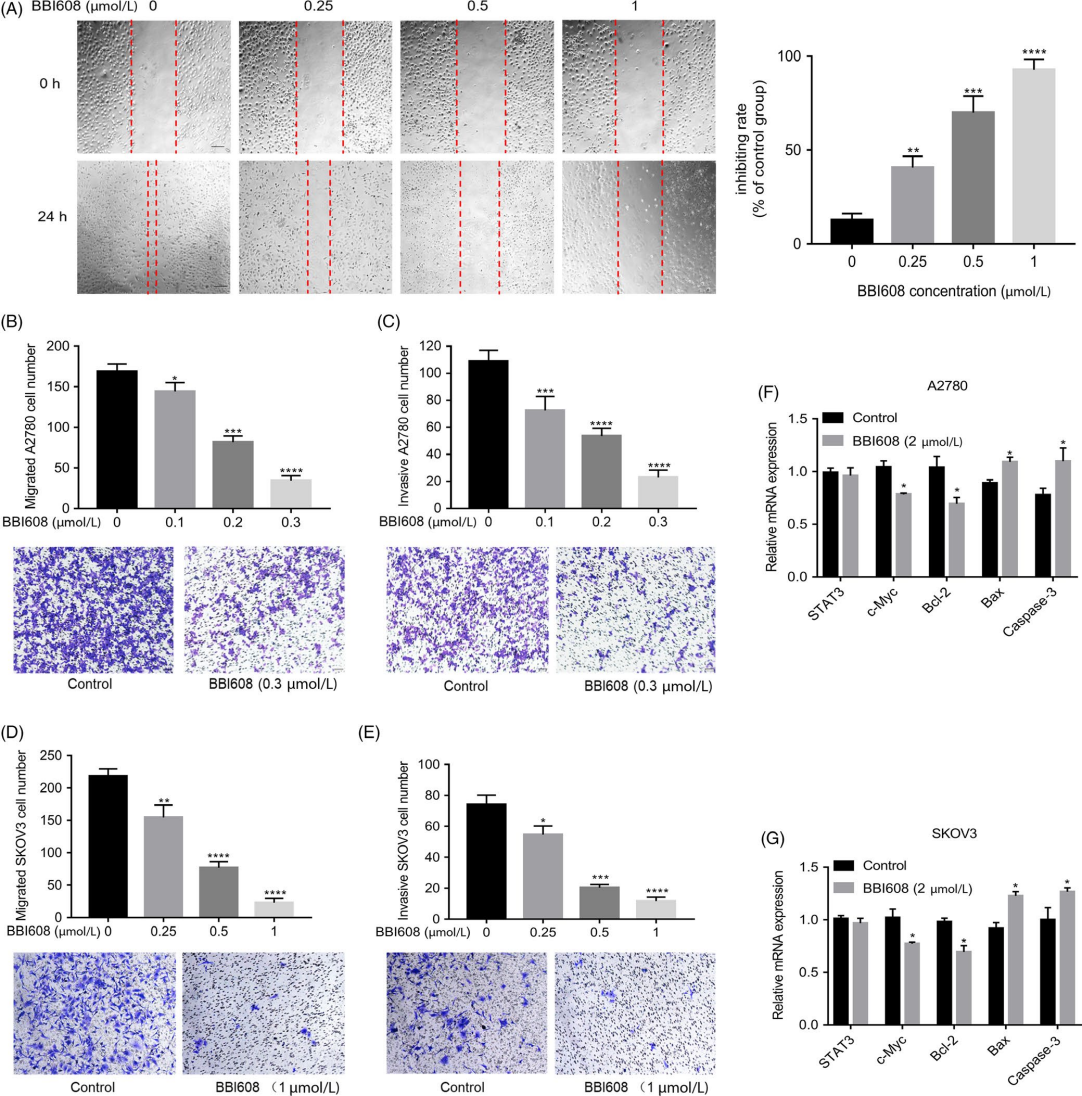
BBI608 inhibits ovarian cancer cell migrative and invasive abilities and pStat3‐dependent apoptosis gene expression. (A) Cell migration capacity of EOC cell SKOV3 was determined by wound healing assay (magnification, ×100). Data are mean ± SD of three independent experiments. (B) and (C) Cell migration and invasion capacity of EOC cell A2780 were determined by transwell assay. The number of migrated and invaded cells was counted, respectively. Data represent means ± SD (n = 3). (D) and (E) Cell migration and invasion capacity of EOC cell SKOV3 were determined by transwell assay. The number of migrated and invaded cells was counted, respectively. Data represent means ± SD (n = 3). (F) and (G) RNA was isolated, reverse transcribed and then analysed by quantitative PCR for mRNA expression of genes related to ovarian cancer cells apoptosis, with GAPDH as control. *P < .05; **P < .01; ***P < .001, ****P < .0001

We then examined the apoptosis‐inducing effect of BBI608 with the use of the Annexin V/PI dual‐labelling tool by FCM. Both EOC cell lines exhibited a dose‐dependent upregulation in apoptosis after 24‐hour treatment with BBI608 (Figure 3A,C). When the A2780 cells underwent the treatment with 0.3 µM BBI608, the apoptosis rates were 17.7%, but the apoptosis rate rose to 42.09% when cells underwent the treatment with 0.5 µM BBI608. BBI608 at 0.5 µM induced significant apoptosis in A2780 cells, while the same concentration induced less apoptosis in SKOV3 cells (9.24%). Besides, apoptotic cells’ number was counted using NoVo Express Software, and BBI608 increased the number of apoptotic cells in manner relying on dose (*P* < .0001) (Figure 3B,D). Meanwhile, we observed treatment with BBI608 for 24 hours decreased the levels of pStat3, Bcl‐2 and Mcl‐1 in a dose‐dependent manner, and the expression of Bax significantly increased (Figure 3E,G). Furthermore, the RT‐qPCR results also showed significantly decreased mRNA expression of c‐Myc and Bcl‐2 and increased levels of Bax and caspase‐3 in BBI608‐treated ovarian cancer cells (Figure 4F,G, *P* < .05).

### 3.4 BBI608 enhances the induction of apoptosis of EOC cells by paclitaxel and downregulates the expression of various proteins involved in apoptosis

Paclitaxel is now a major mitotic inhibiting agent for cancer chemotherapy. Our experiments were designed to determine whether BBI608 is able to promote cell death triggered by paclitaxel. First, we studied the apoptosis of A2780 and SKOV3 cells induced by different concentrations of BBI608 and paclitaxel for 24 h. As shown in Figure 3A,C, BBI608 evidently promoted the apoptotic influences exerted by paclitaxel in A2780 and SKOV3 cells. Next, we found that paclitaxel alone at suboptimal concentrations slightly affected the expression levels of pStat3, Bcl‐2 and Bax proteins in both cells. Nevertheless, cells treated with combined use of BBI608 and paclitaxel resulted in a significant decrease in the expressions of pStat3 and Bcl‐2 and an increase in Bax and cleaved caspase‐3 protein levels (Figure 3F,H). Overall, these results indicated that BBI608 and paclitaxel combination treatment could increase apoptosis in EOC cells compared to either drug alone.

### 3.5 BBI608 suppresses ovarian cancer cell migration and invasion

Epithelial ovarian cancer metastasis is the predominant contributor to mortality associated with cancer, and the ability of cancer cells to invade and migrate is vital to cancer progression and metastasis.^2^ Therefore, to determine whether BBI608 could inhibit ovarian cancer cells migration and invasion, we performed wound healing assays and transwell migration and invasion assays on EOC cell lines. As shown in Figure 4A, after 24 hours, fewer migrating SKOV3 cells were observed as the drug concentration increased. The inhibitory rates of BBI608 on migration were 40.7%, 69.8% and 92.8% at concentrations of 0.25, 0.5 and 1.0 µM, separately. Results of wound healing assay suggested that treatment with BBI608 caused a decrease in cell migration ability as affected by dose; in the transwell assay, we achieved similar results (Figure 4B,D). In addition, transwell assays showed that downregulation of pStat3 levels by BBI608 remarkably impaired the invasive abilities of A2780 cells and SKOV3 cells (Figure 4C,E). Taken together, the results of the wound healing and transwell assays indicated that BBI608 markedly inhibited the migratory and invasive abilities of A2780 and SKOV3 cells in a manner relying on concentration.

### 3.6 BBI608 suppresses the growth of human epithelial ovarian tumours in vivo and inhibits the activation of Stat3 in tumour tissues

In order to ascertain the anti‐tumour influences of BBI608 on SKOV3 ovarian cancer cells in vivo, a subcutaneously implanted xenograft nude mouse model was employed. SKOV3 tumour‐bearing mice were either untreated (control) or treated with vehicle or BBI608 at a dose of 40 mg/kg. The results (Figure S3A,B) show that in comparison with the vehicle‐treated and control group, treatment with 40 mg/kg of BBI608 for 21 days resulted in a significant reduction in tumour volume and tumour weight of SKOV3 xenograft mice (*P* < .01). In addition, there is no significant difference in body weight among the three groups, suggesting that BBI608 exerted no noticeable toxicity on nude mice during the 21 days of treatment (Figure S4). According to the mentioned results, BBI608 had potent anti‐tumour efficacy in vivo in a human ovarian cancer xenograft model. To further explore the mechanisms by which BBI608 caused SKOV3 tumour growth inhibition in vivo, we performed immunohistochemical analysis on tumour tissues. As Figure S3C shows, when compared with vehicle‐treated group, BBI608 caused a marked reduction in the number of Ki67‐positive cells but the number of cleaved caspase‐3‐positive cells displayed significant upregulation (Figure S3E,F). The results indicated that the anti‐proliferative activity of BBI608 in vivo complied with the in vitro results that BBI608 inhibited SKOV3 cancer cell apoptosis induction and proliferation. Furthermore, immunohistochemical staining of tumour tissues from BBI608‐treated mice displayed a noticeably fewer pStat3‐positive cells than in tumour tissues from vehicle‐treated mice (Figure S3D).

### 3.7 BBI608 potentiates the anti‐tumour effects of paclitaxel in an intraperitoneal ovarian cancer xenograft model

**Figure 5:**
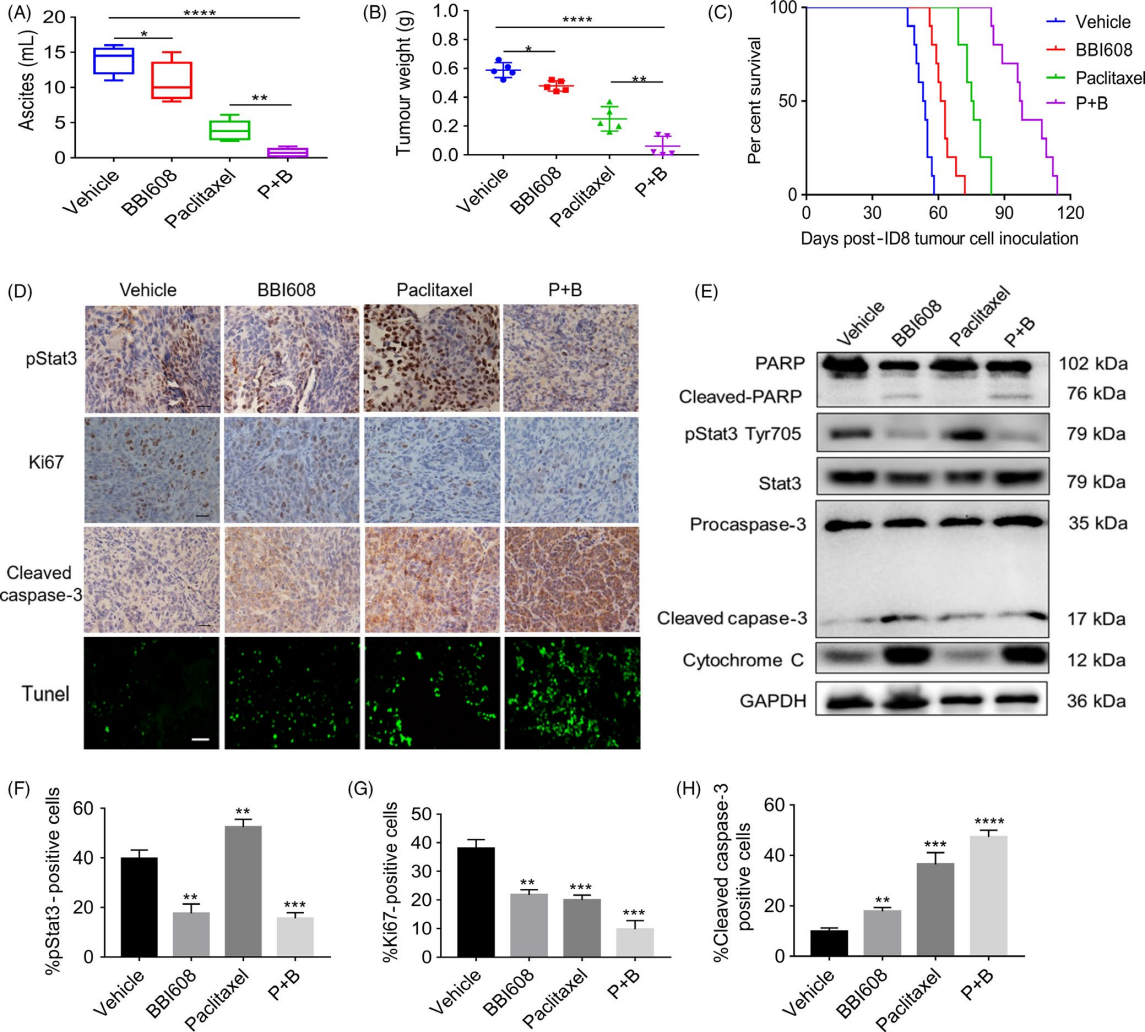
BBI608 enhanced the anti‐tumour effect of paclitaxel in a model of peritoneal tumour of ovarian cancer. (A) Ascites were collected and the volume was recorded immediately after the mice sacrificed. Each data point represents the mean ± SD of 5 mice. (B) Total tumour burden obtained from each mouse was calculated at the day of sacrificed (n = 5/group). (C) Kaplan‐Meier curve depicting the survival of ID8 tumour‐bearing mice (n = 10/group). (D) Immunohistochemistry staining of tumour sections for the expression of pStat3, Ki67 and cleaved caspase‐3 was performed as described in BBI608 either alone or in combination with paclitaxel‐treated tumour samples, as compared with vehicle group (magnification, ×400). TUNEL staining of tumour tissue sections from ID8 tumour‐bearing mice which received vehicle, BBI608, paclitaxel and the combination drugs treatment (magnification, ×400). (E) Western blot analysis showed the inhibition of pStat3 (Tyr705), by BBI608 either alone or in combination with paclitaxel‐treated groups in whole‐cell extracts from mice tissue. Antibodies against procaspase‐3, cleaved caspase‐3, PARP and cleaved PARP were used to detect protein expression involved in apoptotic pathway with GAPDH antibody to verify equal protein loading. (F), (G) and (H) Immunohistochemical staining analysed the expression of pStat3, Ki67, cleaved caspase‐3 proteins. *P < .05, **P < .01; ***P < .001; ****P < .0001

We also tested the anti‐tumour potential of BBI608 and paclitaxel either independently or jointly in an intraperitoneal implanted mouse model of EOC using ID8 cells. For resembling human ovarian cancer cells remaining in the abdominal cavity after surgery and providing relevant insight into treatment strategies, we built the intraperitoneal mouse model. Based on previous work from our subcutaneous xenograft model, a 40 mg/kg dose of BBI608 was administered orally to tumour‐bearing mice. As taxane‐containing agents are the standard of care for ovarian cancer patients, the comparison between paclitaxel (12 mg/kg, intraperitoneally (i.p.) once weekly) and BBI608 was drawn. Treatment was started 10 days after tumour cell implantation, and mice were sacrificed when the body weight of mice in the vehicle control group increased by 50%. We found that compared with the vehicle control, treatment with 40 mg/kg BBI608 alone elicited a marked reduction in the accumulation of ascites (*P* < .05). Ascites volume was also significantly decreased in paclitaxel monotherapy compared with the vehicle control (*P* < .01). The combined use of two drugs was more effective in reducing the tumour ascites (Figure 5A). The average weight of the debulked tumours (Figure 5B) in the BBI608 and paclitaxel combination group was noticeably less than the paclitaxel alone or BBI608 alone groups.

We then assessed the effect exerted by BBI608 and paclitaxel on constitutive nuclear expression of pStat3 in EOC tumour tissues through immunohistochemical analysis and found that compared with the vehicle control, BBI608, either independently or jointly with paclitaxel, inhibited Stat3 activation in a moderate manner by inhibiting phosphorylation at Tyr705 (Figure 5D,F). These results suggested that combination treatment of BBI608 and paclitaxel basically inhibited the activation of constitutive Stat3. However, treatment with paclitaxel alone resulted in a marked increase in nuclear pStat3 levels. The results of immunohistochemical analysis also demonstrated that decreased levels of pStat3 in tumour tissues treated with BBI608 and paclitaxel correlate with the appearance of cleaved caspase‐3 (a biomarker of apoptosis) (Figure 5H). Compared with the control group, both BBI608 and paclitaxel alone had obvious inhibitory effect on the expression of Ki67 in tumour tissues, and the combined effect of these two compounds was significantly more effective (Figure 5G).

### 3.8 BBI608 inhibits Stat3 activation in EOC tumour tissues and activates the apoptotic cascade

The activation of Stat3 has been suggested to regulate the expressions of many different gene products participating in anti‐apoptotic activity (Bcl‐2, Bax, Mcl‐1 and cleaved caspase‐3) in vitro. Thus, we sought to determine whether the apoptotic pathway is involved in BBI608 and paclitaxel treatment in tumour tissues by performing a Western blot analysis. Consistent with the in vitro results, the BBI608 and paclitaxel combination were more feasible in downregulating pStat3 proteins’ overexpression, while paclitaxel alone seemed to upregulate the level of pStat3 (Figure S7). In addition, the activation of PARP and caspase‐3 was further enhanced by co‐treatment of BBI608 and paclitaxel in EOC tumour tissues, along with increased levels of cytochrome C (Figure 5E). According to the mentioned results, the combination of BBI608 and paclitaxel can activate the apoptotic cascade in tumour tissues, which then leads to reduced tumour burden.

### 3.9 Toxicity evaluation

As mentioned above, BBI608 treatment for 21 days suggested no adverse effects on gross measures (eg body weight loss, skin ulceration, diarrhoea and toxic death). In order to further evaluate the in vivo safety of BBI608, we determined whether BBI608 could cause abnormalities in the blood system and blood biochemical analyses of ALT, ALT, TP, TG, CK and CREA were conducted. The data in Figure S5 show that mice treated with BBI608 did not display noticeable differences in any of these parameters compared with mice in the vehicle group. There were some fluctuations among each group, but all of them ranged within normal values. Moreover, the toxicity of BBI608 to mouse heart, liver, spleens, lung and kidney was further assessed by H&E staining analysis. No pathologic changes in morphology were observed between the BBI608‐treated group and the vehicle (Figure S6), revealing no signs of adverse effects on the heart, liver, spleen, lung and kidney cells in vivo.

## 4 DISCUSSION

Epithelial ovarian cancer is highly malignant and shows considerable metastatic potential, prompting the development of new potential drug candidates to prevent tumour metastasis and suppress tumour growth. Stat3, which can be triggered by cytokines and growth factors, is persistently activated in many types of cancer. It is not only involved in cancer development and progression^21^, ^22^ but also appeared to be associated with the prognosis of cancer patients. Dolled‐Filaret et al^23^ detected constitutive nuclear expression of pStat3 in node‐negative breast cancer, and patients with pStat3‐positive tumours have remarkably enhanced survival at both short‐term (5‐year survival) and long‐term (20‐year survival). In another study, Stat3 suggested mixed nuclear and cytoplasmic staining in head and neck squamous cell carcinoma, and the high nuclear Stat3 expression contributed to an increase in progression‐free survival by 42.2 months.^24^ Conversely, the expression of pStat3 in tumour tissues was found to be related to poor prognoses of head and neck squamous cell carcinoma, renal cell cancer, colorectal cancer and gastric cancer.^8^, ^25^, ^26^, ^27^ In this study, a significant correlation between the nuclear pStat3 expression and a low prognosis in EOC patients was detected. This was confirmed by several previous studies in ovarian cancer tissues, which showed that patients who had tumours with high nuclear expression of pStat3 had poorer survival rates and shorter survival than women who had tumours with low nuclear pStat3 expression.^8^, ^9^ These results suggest that targeting Stat3 presents a feasible strategy to improve the outcomes of patients with ovarian cancer.

Constitutive Stat3 activation has intrinsic consequences on the tumour cell as well as effects within the extracellular matrix (ECM) and stromal cells of the tumour microenvironment, thus resulting in increased tumour cell proliferation, motility, survival and invasiveness, as well as tumour‐promoting activities of angiogenesis and evasion of tumour‐suppressing immunity.^28^ Based on its common activation in ovarian carcinoma and its extensive cellular activities, Stat3 has become an attractive therapeutic target. This study primarily aimed to ascertain whether BBI608 exerts its anti‐cancer effects by modulating negative regulators of the Stat3 signalling pathway in epithelial ovarian cancer cells. We detected for the first time that BBI608 decreased the levels of Stat3 phosphorylation at tyrosine residue 705 in SKOV3 human epithelial ovarian cancer cells, and the phosphorylation Stat3 has exhibited close relation to tumour cells’ proliferation and transformation. Furthermore, according to in vitro studies, BBI608 combined with paclitaxel can inhibit ovarian cancer cell vitality at low micromole concentrations. Inhibition of pStat3 with BBI608 also effectively downregulated the colony‐forming ability of ovarian cancer cells. Constitutive activation of Stat3 has been known to trigger resistance to apoptosis,^29^ possibly by upregulation of survivin, IAP1/2, Bcl‐xl and Bcl‐2 expression.^30^ The results of the FCM and observed protein expression levels from the Western blot assays signified that BBI608 induced apoptosis in ovarian cancer cells in a dose‐dependent manner, as verified by the increase in Bax and cleaved caspase‐3 and the reduction in Bcl‐2 and Mcl‐1. According to these mentioned results, the anti‐tumour effects of BBI608 are related to the proliferation suppression and induction of apoptosis in ovarian cancer cells.

Ovarian cancer progresses towards progressively malignant behaviour in tumorigenic and metastatic stages. During metastasis, tumour cells will leave the primary tumour in the ovary and metastasize to abdominal organs (omentum, spleen, lymph node and liver), in which they develop secondary tumours and malignant ascites.^3^, ^31^, ^32^ Recent studies have suggested that constitutively activated Stat3 signalling pathway is critical to the invasion and metastasis of malignant tumours. Meanwhile, the expression of pStat3 was confirmed to be significantly correlated with tumour stage, differentiation and presence of lymph node metastasis.^8^, ^33^ Moreover, recent published in vivo studies have shown that Stat3 knockdown reduces ovarian tumour growth and metastatic potential, revealing that Stat3 activation is required by ovarian cancer progression and metastasis.^34^, ^35^ Infiltration of surrounding tissues and distant metastasis are two crucial malignant tumour hallmarks, evidently affecting the prognosis and therapy success rate of cases. Therefore, by wound healing and transwell assays, the migration and invasion abilities of ovarian cancer cells were ascertained. According to the results here, in addition to its pro‐apoptotic and anti‐proliferative functions, BBI608 showed significant anti‐invasion and anti‐migration activities in ovarian cancer cells (Figure 4). Overexpression of Stat3 and IL‐6 could induce the expression of HIF‐1α and VEGF.^36^, ^37^ Blockade of pStat3 can reduce the expression of HIF‐α, VEGF, MMP2 and MMP‐9 and inhibit the proliferation and invasion of cancer cells.^38^ Our results demonstrated that inhibition of Stat3 decreases the metastatic potential of ovarian cancer cells.

To explore the anti‐tumour activity of BBI608 and its related mechanism in vivo, we used a well‐established subcutaneous SKOV3 tumour model in female BALB/c mice. The results showed that BBI608 inhibited tumour growth by 73.4% at a dose of 40 mg/kg. Furthermore, we identified that compared with the vehicle treatment, BBI608 (a potent small‐molecule inhibitor of Stat3) reduced the expression of the proliferation marker Ki67 and increased the expression of cleaved caspase‐3 in tumour cells by inhibiting Stat3 activation in ovarian tumour cells. In addition, increasing evidence suggests that pStat3 plays an important role in upregulating VEGF gene expression and triggering tumour angiogenesis in physiological and pathological environments.^39^ Inhibition of Stat3 can suppress tumour angiogenesis, as detected in this study in tumour tissues treated with BBI608 as well (data not shown).

Though paclitaxel has been extensively applied to treat advanced EOC, this drug has severe side effects, and cases frequently have adaptive chemoresistance and consequent tumour recurrence. However, paclitaxel has shown synergistic interaction with various classes of targeted therapeutic agents (eg anti‐VEGF inhibitors and PARP inhibitors) and is currently being assessed in adjuvant and neo‐adjuvant treatment settings for ovarian cancer.^40^ Interestingly, BBI608 can enhance paclitaxel sensitivity in A2780 and SKOV3 cells at a low concentration of 1nM in a synergistical manner. The combination treatment potentiated paclitaxel‐induced apoptosis by downregulating various Stat3‐regulated gene products. Besides, the combined treatment of BBI608 and paclitaxel noticeably hindered tumour growth in an intraperitoneal xenograft ovarian cancer model, evidently activated the apoptotic pathway, decreased the levels of pStat3 in tumour tissues and prolonged survival time of tumour‐bearing mice (Figure 5C).

## 5 CONCLUSION

The results here demonstrate initially that BBI608 can significantly suppress the persistent Stat3 activation signalling pathway and can promote the effects exerted by paclitaxel on epithelial ovarian cancer in vivo and in vitro by downregulating gene products that mediate tumour cell proliferation, metastasis, invasion and survival. Future studies should focus on elucidating Stat3’s activating mechanism and its effects on downstream targets and on exploring the function of BBI608 in controlling relapse and metastasis of ovarian cancer because this compound could be vital in the clinic.

## CONFLICT OF INTERESTS

The authors declare that they have no competing interests.

## AUTHORS’ CONTRIBUTIONS

All authors read and approved the final manuscript. XWW and XZ conceived and designed the experiments; HYL and YPQ performed all the experiments and HYL drafted the manuscript; XW and RYP participated in the statistical analyses; XWW revised the manuscript and provided important suggestions.

## ETHICS APPROVAL AND CONSENT TO PARTICIPATE

This study was approved by the institutional review board/ethics committee of the Shanghai Outdo Biotech (Permit Number: YBM0502).

## Data Availability

The datasets used and/or analysed during the current study are available from the corresponding author on reasonable request.
