# Supplementary material for: Targeted activation of Stat3 in combination with paclitaxel results in increased apoptosis in epithelial ovarian cancer cells and a reduced tumour burden
Source: Cell Prolif. 2019 Nov 28;53(1):e12719. doi: 10.1111/cpr.12719 (PMC6985655; doi:10.1111/cpr.12719)
Supplement: Supplementary file 1 [file CPR-53-e12719-s001.docx]

**Targeted activation of Stat3 in combination with paclitaxel results in increased apoptosis in epithelial ovarian cancer cells and a reduced tumour burden**

Hongyi Li^a^, Yanping Qian^a^, Xi Wang^a^, Ruyu Pi^a^, Xia Zhao^a,^ *, Xiawei Wei^b,^ *

^a^ Department of Gynecology and Obstetrics, Development and Related Disease of Women and Children Key Laboratory of Sichuan Province, Key Laboratory of Birth Defects and Related Diseases of Women and Children, Ministry of Education, West China Second Hospital, Sichuan University, Chengdu, 610041, P. R. China.

^b^ Lab of Aging Research and Nanotoxicology, State Key Laboratory of Biotherapy, West China Hospital, Sichuan University and Collaborative Innovation Center, No. 17, Block 3, Southern Renmin Road, Chengdu, Sichuan 610041, P.R. China.

**Running title: Stat3 inhibitor synergizes with paclitaxel in EOC**

**Corresponding authors at:** Xia Zhao and Xiawei Wei

NO.1 Keyuan Street Road 4, Gaopeng Street, Chengdu, Sichuan Province, 610041, China.

**Tel/Fax:** 86-28-85502796

**E-mail addresses:** [xiazhaoscu@126.com](mailto:xiazhaoscu@126.com) (X. Zhao); [xiaweiwei@scu.edu.cn](mailto:xiaweiwei@scu.edu.cn) (X. Wei).

Supplementary Data

**Supplementary Table 1:** RT-qPCR primers used in this study.

**Supplementary Figure 1:** The drug IC50 value of A2780, ID-8 and SKOV3 cells treated with different concentrations of BBI608/BBI608+paclitaxel (1nM) 24h.

**Supplementary Figure 2:** Histological analysis of the colony number showed that the colony formation of SKOV3 cell line were dramatically decreased after treated with combined paclitaxel and BBI608.

**Supplementary Figure 3:** BBI608 suppressed the tumor growth on ovarian cancer mouse xenograft models.

**Supplementary Figure 4:** No significant difference was found in body weight between the vehicle/control group and the BBI608-treated group.

**Supplementary Figure 5:** There were no significant differences between the vehicle-treated group and the BBI608-treated groups in blood biochemical analysis of toxicity test of ALT, AST, TP, TG, CK and CREA.

**Supplementary Figure 6:** BBI608 did not cause obvious pathologic abnormalities in normal tissues.

**Supplementary Figure 7:** Relative gray values of the pStat3 protein.

| Gene name | sequence |
| --- | --- |
| GAPDH | Forward GGTCGGAGTCAACGGATTTGGTCG  Reverse CCTCCGACGCCTGCTTCACCAC |
| STAT3 | Forward CTGGCCTTTGGTGTTGAAAT  Reverse AGGGCGAGGACCATAGAGG |
| c-Myc | Forward GCGTCCTGGGAAGGGAGATCCGGAGC  Reverse TTGAGGGGCATCGTCGCGGGAGGCTG |
| Bcl2 | Forward GATCCTCGAGATGGCGCACGCTGGGAGAAC  Reverse GATCGGATCCTCATGGCTGAGCGCAG |
| Bax | Forward CCCGAGAGGTCTTTTTCC  Reverse GCCTTGAGCACCAGTTTG |
| Caspase3 | Forward ACATGGGAGCAAGTCAGTGG  Reverse CGTCCACATCCGTACCAGAG |

**Supplementary Table 1:** RT-qPCR primers used in this study.


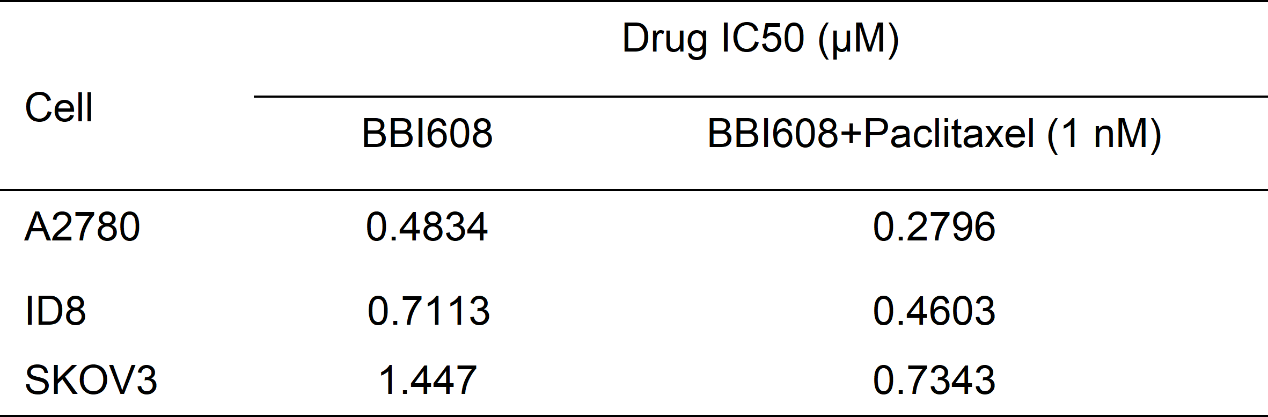


**Supplementary Figure 1:** The drug IC50 value of A2780, ID-8 and SKOV3 cells treated with different concentrations of BBI608/BBI608+paclitaxel (1nM) 24h.


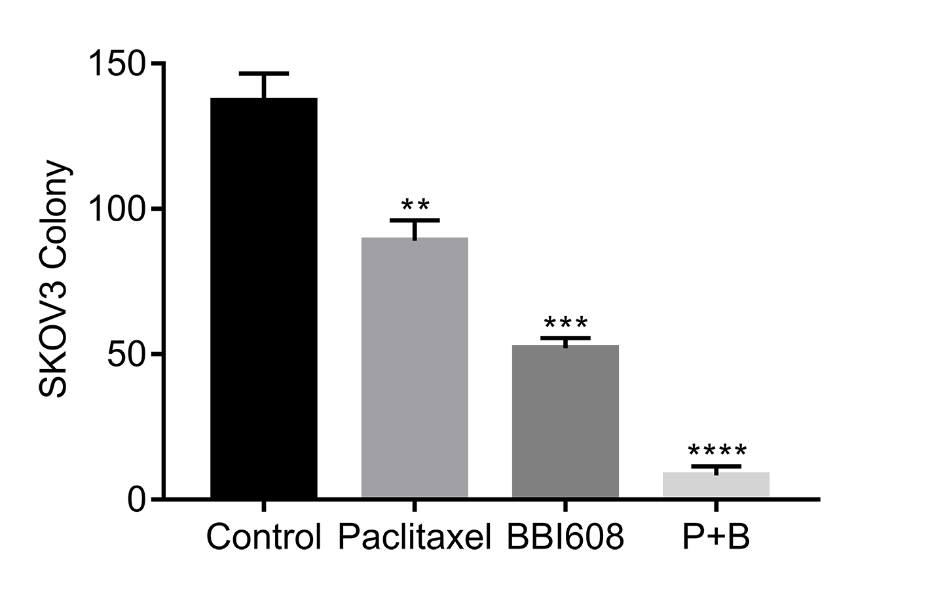


**Supplementary Figure 2:** Histological analysis of the colony number showed that the colony formation of SKOV3 cell line were dramatically decreased after treated with combined paclitaxel (1nM) and BBI608 (1μM) for 24 h (**P < 0.01; ***P < 0.001; ****P < 0.001; n = 3).


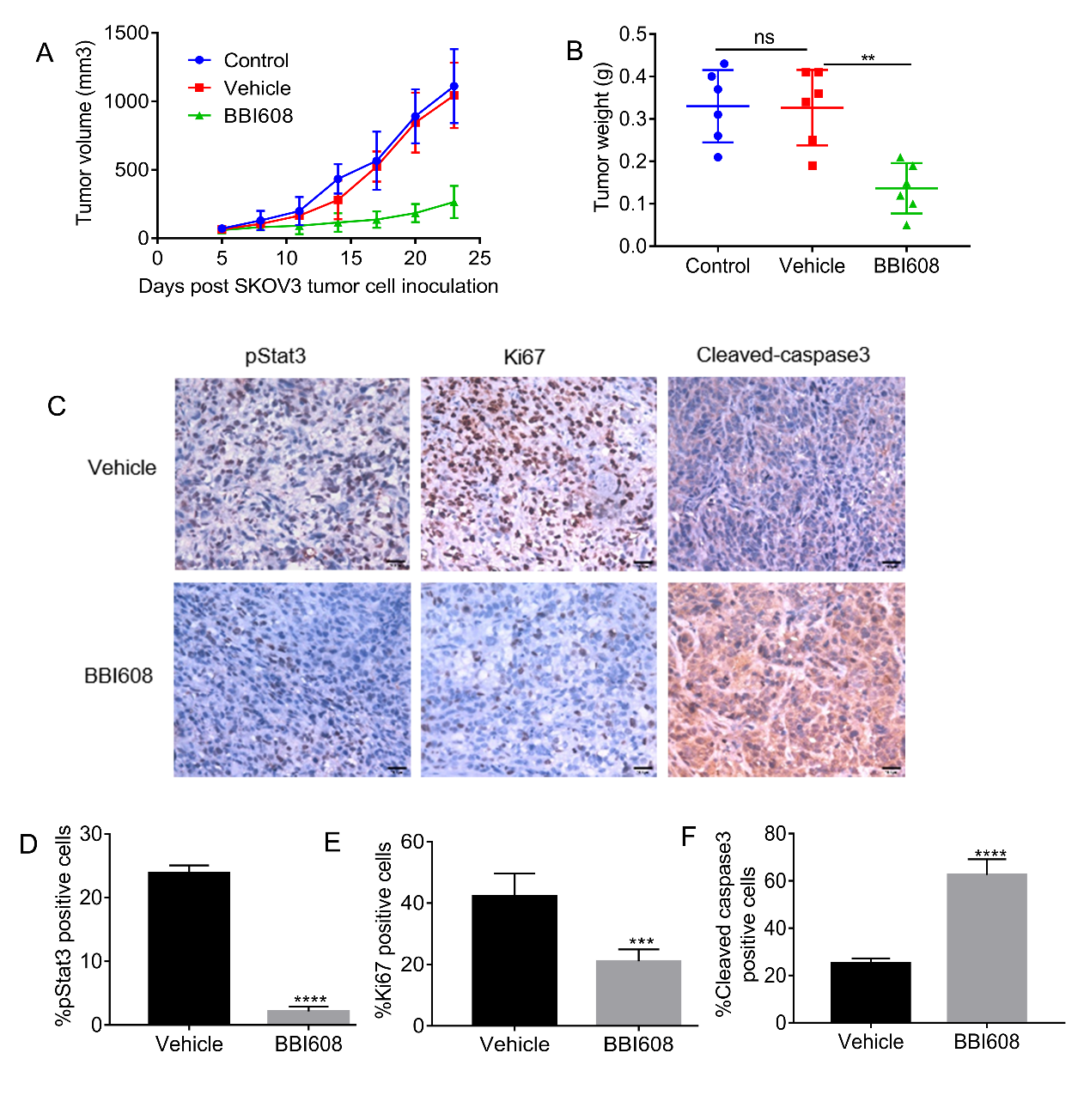


**Supplementary Figure 3:** BBI608 suppressed the tumor growth on ovarian cancer mouse xenograft models.

(A) The mice treated with BBI608 showed marked reduction in tumor growth compared with the mice treated with vehicle or no treatment group (control) (n = 6/group). (B) The weight of the tumors was significantly decreased in BBI608-treated mice than in vehicle-treated mice (control). (C) Immunohistochemistry staining of paraffin-embedded SKOV3 tumor sections for the expression of pStat3, Ki67 and cleaved-caspase3, (magnification, ×400). (D), (E) and (F) Statistical histogram of the positive expression of pStat3, Ki67 and Cleaved-caspase3 proteins. ns not significant; **p<0.01; ***p<0.001; ****p<0.0001


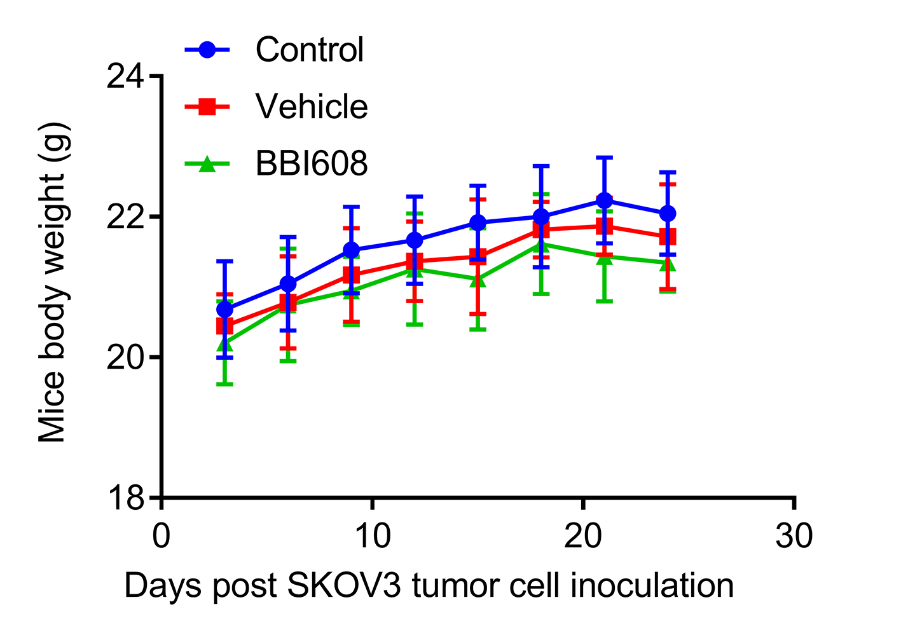


**Supplementary Figure 4:** No significant difference was found in body weight between the vehicle/control group and the BBI608-treated group.


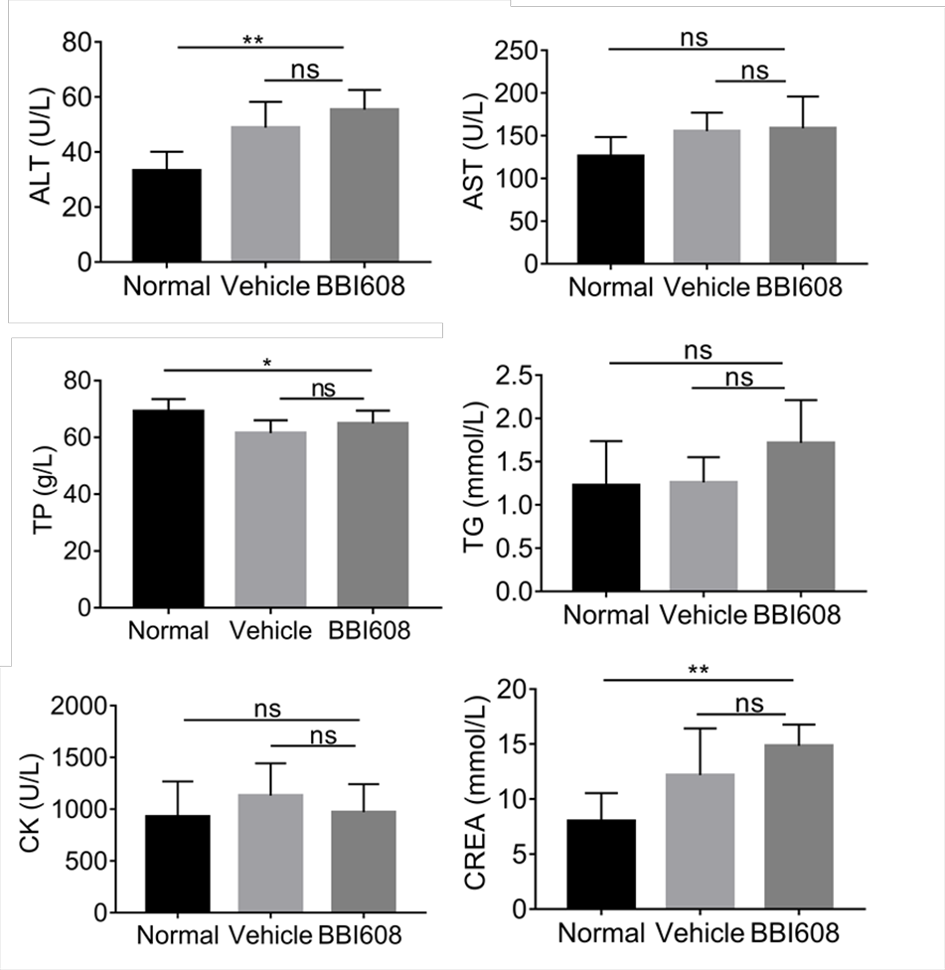


**Supplementary Figure 5:** There were no significant differences between the vehicle-treated group and the BBI608-treated groups in blood biochemical analysis of toxicity test of ALT, AST, TP, TG, CK and CREA.


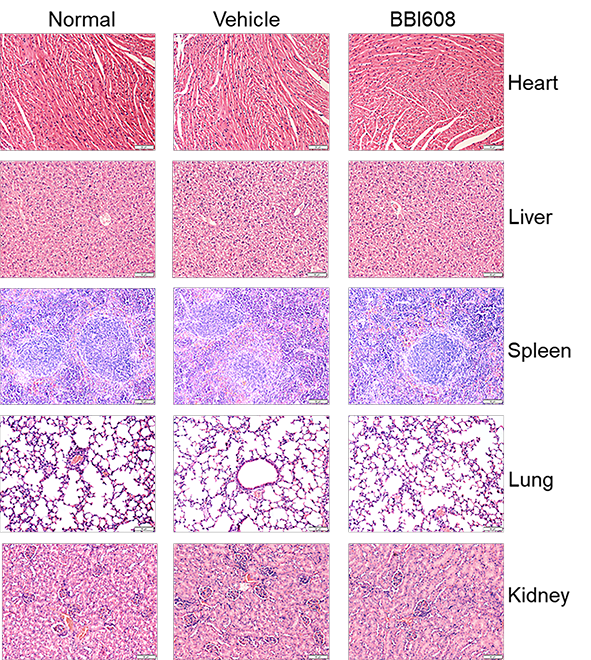


**Supplementary Figure 6:** BBI608 did not cause obvious pathologic abnormalities in normal tissues. H&E staining of paraffin-embedded sections of the heart, liver, spleen, lung and kidney (magnification, ×200).


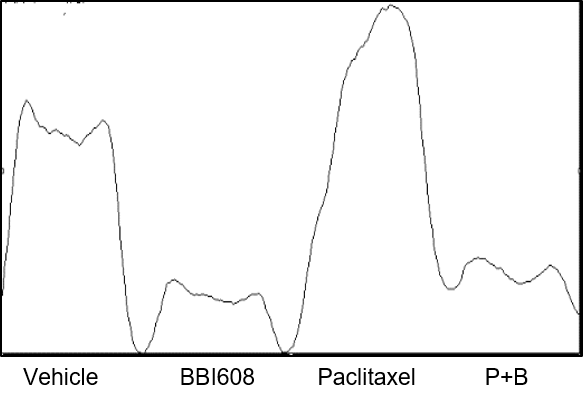

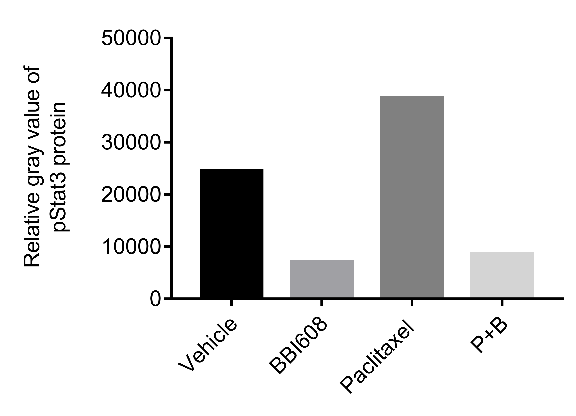


**Supplementary Figure 7:** Relative gray values of the pStat3 protein.
